# Adoptive T Cell Immunotherapy for Cancer

**DOI:** 10.5041/RMMJ.10179

**Published:** 2015-01-29

**Authors:** Karlo Perica, Juan Carlos Varela, Mathias Oelke, Jonathan Schneck

**Affiliations:** 1 Department of Biomedical Engineering, Johns Hopkins School of Medicine, Baltimore, MD, USA;; 2 Institute of Cell Engineering, Johns Hopkins School of Medicine, Baltimore, MD, USA;; 3 Sidney Kimmel Comprehensive Cancer Center, Johns Hopkins School of Medicine, Baltimore, MD, USA;; 4 Department of Pathology, Johns Hopkins School of Medicine, Baltimore, MD, USA;; 5 Department of Oncology, Johns Hopkins School of Medicine, Baltimore, MD, USA; 6 Department of Medicine, Johns Hopkins School of Medicine, Baltimore, MD, USA

**Keywords:** Adoptive T cell transfer, cancer immunotherapy, T cell engineering

## Abstract

Harnessing the immune system to recognize and destroy tumor cells has been the central goal of anti-cancer immunotherapy. In recent years, there has been an increased interest in optimizing this technology in order to make it a clinically feasible treatment. One of the main treatment modalities within cancer immunotherapy has been adoptive T cell therapy (ACT). Using this approach, tumor-specific cytotoxic T cells are infused into cancer patients with the goal of recognizing, targeting, and destroying tumor cells. In the current review, we revisit some of the major successes of ACT, the major hurdles that have been overcome to optimize ACT, the remaining challenges, and future approaches to make ACT widely available.

## INTRODUCTION

Historically, there have been three pillars of cancer treatment: surgery, chemotherapy, and radiotherapy. In recent years, immunotherapy has emerged as a possible fourth pillar, targeting cancer not by its anatomic location or propensity to divide, but by the inherent mechanisms the immune system uses to distinguish between healthy and pathologic tissue. Adoptive T cell therapy (ACT) is one stone in this new pillar, a potentially powerful approach to cancer treatment that relies on the infusion of tumor-specific T cells.

From a theoretical standpoint, cancer immunotherapy using T cells has long been of interest. Adaptive immunity has numerous beneficial properties that make it amenable for cancer treatment: 1) T cell responses are specific, and can thus potentially distinguish between healthy and cancerous tissue; 2) T cells responses are robust, undergoing up to 1,000-fold clonal expansion after activation; 3) T cell response can traffic to the site of antigen, suggesting a mechanism for eradication of distant metastases; and 4) T cell responses have memory, maintaining therapeutic effect for many years after initial treatment.

Despite this theoretical interest, cancer immunotherapy could not proceed until it was established that immunity could distinguish tumor from healthy tissue.^1^ Unlike microbial pathogens, tumors are fundamentally “self,” and belief in immune recognition of cancer has waxed and waned since the cancer immunosurveillance hypothesis was first proposed. The identification of human tumor-associated antigens (TAAs)^2^ was the culmination of a renewed interest in tumor immunology spurred by tumor transplant models in the mouse^3^ and provided definitive proof that specific anti-tumor responses could be generated under the right conditions.

The next hurdle to be addressed was the identification of a readily accessible pool of tumor-specific lymphocytes. Using modern techniques, T cells with anti-tumor cytotoxic activity can be identified in tumor samples of up to 80% of melanoma patients^4^ (but less frequently in other cancers). It is now clear that T cell infiltration and inflammation are “hallmarks” of cancer.^5^ Still, the immune surveillance hypothesis remains controversial, and whether tumor-infiltrating lymphocytes (TILs) are spectators to cell death and dysfunction or actively mediating rejection of human cancers is open to debate. Furthermore, the very coexistence of tumor-specific T cells and large tumors casts doubt on the effectiveness of these responses in cancer eradication. However, while the question of whether immunity *does* control cancer remains a matter of some debate, adoptive cell therapy has conclusively demonstrated that, under the right therapeutic conditions, ACT *can* eradicate tumor.

## CANCER, IMMUNITY, AND ADOPTIVE CELL THERAPY

Based on their use in ACT, we will focus here on the tumor immunology of T cells specifically. To mount an effective and targeted response, T cells must be able to recognize and target specific antigens presented in the context of major histocompatibility complex (MHC) proteins on the tumor that are not present or are poorly expressed on healthy tissue.

**Figure 1.**
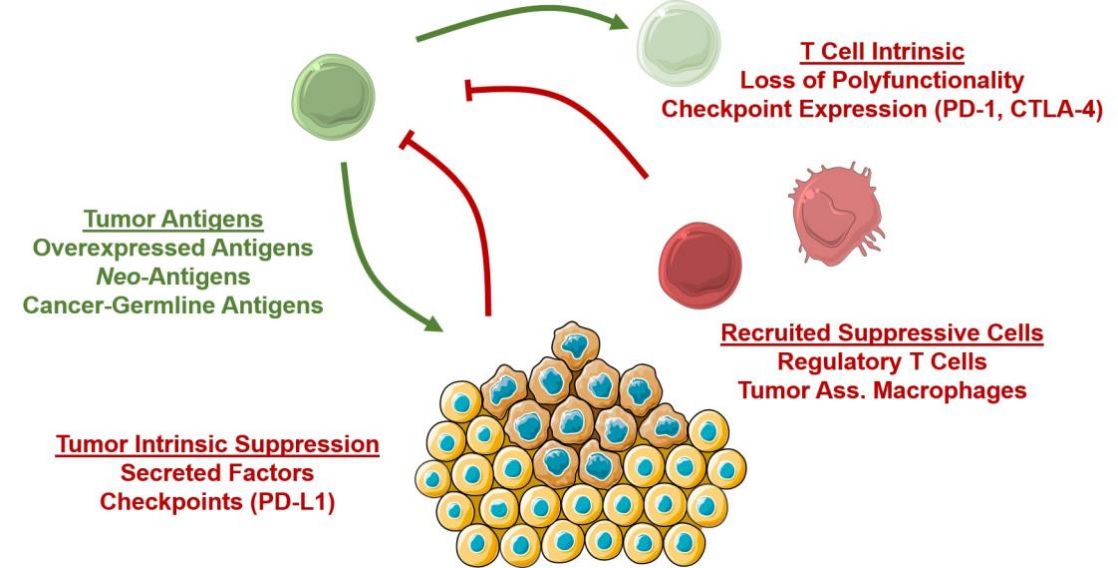
**T Cell Anti-Cancer Responses.** Tumor-specific T cells (green) can recognize over-expressed antigens, neo-antigens derived from germline mutations, or so-called cancer germline antigens expressed *de novo* during carcinogenesis. However, several processes exist to suppress anti-cancer responses. T cell-intrinsic mechanisms such as loss of functionality and expression of checkpoint proteins (PD-1, CTLA-4) lead to T cell exhaustion. Tumor-intrinsic mechanisms include secretion of suppressive factors such as TGF-B, or expression or checkpoint ligands. Furthermore, tumors recruit suppressive cells such as regulatory T cells and tumor-associated macrophages that further inhibit T cell responses.

Tumor-associated antigens (TAAs) were identified by seminal studies in the 1990s which conclusively demonstrated that immune cells could distinguish cancerous from healthy cells.^1^ Tumor-associated antigens can be classified into three major groups (Figure 1)^6^: 1) Antigens over-expressed in tumors which are present on healthy tissue, but are over-expressed in cancer, often because they provide a growth advantage to the cell. These include the melanoma differentiation antigens, derived from differentiation proteins specific to the melanocyte lineage, are over-expressed in melanoma, and are recognized by TILs in many patients. 2) Neo-antigens arising from somatic mutations in cancer. 3) Cancer germline antigens, proteins that are normally expressed on germline cells, which reside in an immunoprivileged site and are thus less vulnerable to autoimmune T cell targeting.

Armed with the knowledge that T cells could target cancer, investigators developed cancer vaccines to activate anti-tumor immunity.^7^ Whether based on proteins, peptides derived from known TAAs, or whole cancer cells modified to enhance their immunogenicity, cancer vaccines were effective at inducing T cell responses but not effective at inducing tumor regression. We now know that both central and peripheral tolerance mechanisms exist that limit productive anti-tumor immunity even when anti-tumor T cells are present in the host (Figure 1). For example, T cells that strongly recognize self-antigens are deleted during thymic development, a process known as central tolerance,^8^ which necessarily limits the avidity of T cells that recognize over-expressed self-proteins. Perhaps even more critical to tumor immune escape are peripheral tolerance mechanisms, which exist to protect host tissues from over-exuberant immune responses that unchecked lead to autoimmunity and are subverted during carcinogenesis. Broadly speaking, these mechanisms can be divided into several categories, including: 1) T cell-intrinsic mechanisms, which include processes such as T cell “exhaustion” that reduce T cell effectiveness in the setting of chronic, low inflammatory processes;^9^ 2) Tumor-intrinsic mechanisms, such as the secretion of immunoregulatory proteins such as TGF-B,^10^ and the expression of checkpoint molecules such as PD-1 that suppress immune responses;^11^ and 3) Recruitment of regulatory cells such as regulatory T cells and myeloid derived suppressor cells^12^ that also suppress immune responses through a variety of overlapping mechanisms. These mechanisms of peripheral tolerance explain how immunogenic tumors such as melanoma exist even in the presence of cytotoxic T cell infiltrates which include tumor-specific cells.

Eliminating these peripheral resistance mechanisms has emerged as a powerful approach to cancer therapy, with “checkpoint blockade” attracting attention based on the results of several successful clinical trials in melanoma.^13^,^14^ Blocking inhibitory molecules such as CTLA-4 and PD-1 (or its receptors) that are expressed on T cells or their receptors (such as PD-L1) expressed on antigen-presenting cells or tumors activates immunity and unleashes immune responses already present in the host.^11^ This technique is non-specific and can also unleash autoimmune T cell responses against healthy host tissue, leading to significant autoimmune toxicities.

**Figure 2.**
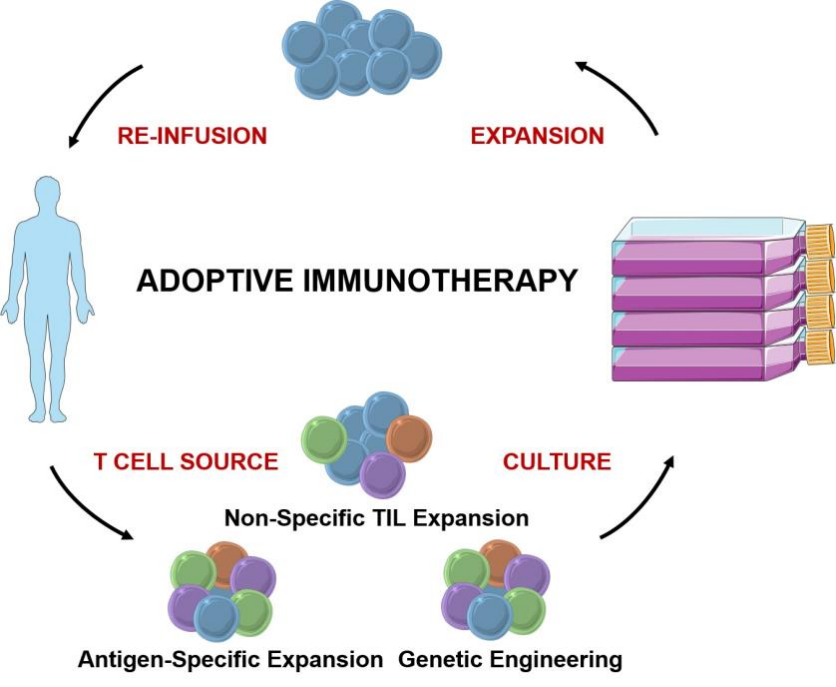
**The Process of Adoptive T Cell Immunotherapy.** T cells are harvested either from tumor (tumor-infiltrating lymphocytes, TILs) or peripheral blood (peripheral blood lymphocytes, PBLs). TILs can be expanded non-specifically since they are preferentially tumor-specific prior to culture. In contrast, tumor specificity must be induced in PBLs, either through antigen-specific expansion or genetic engineering. After several weeks of expansion in culture, tumor-specific T cells can be reinfused into the cancer patient.

Adoptive T cell therapy, in contrast, creates rather than unleashes a productive immune response. Through one of several techniques, T cells are harvested from a patient’s blood or tumor, then stimulated to grow and expand in an *in vitro* culture system (Figure 2). After sufficient *in vitro* expansion, these cells are reinfused into the host, where they will hopefully mediate tumor destruction. Thus, this process is applicable to the vast majority of cancer patients that do not seem to possess a productive anti-cancer response prior to intervention, and therefore at least theoretically will not respond to being “unblocked” by checkpoint inhibitors.

Furthermore, T cell activation *in vitro* physically separates the growing anti-tumor cells from their host and presents an opportunity to manipulate both cells and host in clinically meaningful ways. For example, a critical breakthrough in the efficacy of ACT occurred with the addition of partial myeloablation prior to reinfusion of cultured T cells. Using chemotherapy and radiation, cells that mediate peripheral tolerance such as regulatory T cells can be at least temporarily ablated, along with the rest of the host immune system. In the absence of pharmaceuticals that specifically target these cells, this approach is a more complete and powerful method for eliminating host tolerance and has resulted in significantly improved responses to ACT.^15^–^17^

However, as will be discussed in detail below, this process comes at a high price, quite literally. Adoptive T cell therapy involves the development of an expensive “new drug” for each patient, with T cells grown for weeks in culture and patients hospitalized to receive therapy. The exquisite specificity of T cells is mediated by MHC restriction, and each tumor has a specific set of genomic alterations and mutations that can be targeted. Thus, a given anti-tumor response cannot be exported to the general population. In contrast, therapies like checkpoint inhibitors can be used broadly, and represent a more cost-effective approach for the patients that respond to them. Thus, ACT represents a powerful approach to expanding the benefits of cancer immunotherapy to otherwise non-responsive patients and nonimmunogenic tumors, which at least thus far seem to represent the vast majority of human cancers.

## A HISTORICAL PERSPECTIVE ON ADOPTIVE IMMUNOTHERAPY

While the mechanism of action was not initially understood, allogeneic hematopoietic stem cell transplantation (HSCT) for hematological malignancies represents the earliest adoptive transfer of T cells with anti-cancer activity.^18^ Rather than simply replacing leukemic bone marrow with a healthy transplant, donor cells mediate a graft-versus-tumor effect against allogeneic antigens present on leukemic cells,^19^ which reduces tumor burden and recurrence.^20^ Unfortunately, lack of specificity in the allogeneic responses makes it challenging to separate the graft effect on tumor from the graft effect on host.

The earliest trials of ACT using lymphocytes isolated from cancer samples (also known as tumor-infiltrating lymphocytes, or TILS) were conducted at the surgical branch of the National Cancer Institute (NCI) in Bethesda, Maryland, USA in 1988,^21^ following the demonstration in 1987 that TILs could be cultured with the aid of the lymphotrophic cytokine IL-2 and exhibited cytotoxic activity against cancer cells *in vitro*.^22^ Objective responses by RECIST criteria were observed in 11 of 20 patients with metastatic melanoma, and in 34% of patients of a larger follow-up report in 1994.^23^ Unfortunately, only 5 of the 29 responses were complete, and the median duration of response in these early studies was only 4 months.

A major breakthrough occurred with the addition of lymphodepletion prior to ACT. The benefits of total body irradiation and lymphodepleting chemotherapy were first illustrated in mouse models of B16 melanoma.^24^ The addition of lymphodepletion increased response rates in stage IV melanoma patients to 49%, 52%, and 72% with three sequential protocols of increasing intensity total body irradiation.^15^–^17^ Complete responses were achieved in 20 of 93 patients treated, and 19 of these 20 responses have persisted for at least 5 years. Comparable results have now been achieved outside of the NCI, as shown by a clinical trial that utilized a lymphodepleting chemotherapy regimen with no total body irradiation leading to a response rate of 48% (4 complete, 11 partial).^25^,^26^

While these results represented a stunning breakthrough in melanoma treatment, the protocol could not be applied to patients who lacked readily cultured T cell responses, or to cancers other than melanoma in which TIL culture remained a challenge. For example, both breast and colon cancer tumors have been found to contain TILs; however, their antigen specificities are still incompletely defined, and a significant proportion of those lymphocytes have suppressive rather than anti-tumor activity.^27^,^28^ Recently, sufficient TILs were isolated from cholangiocarcinoma patients to induce remission, but responses to epithelial cancers remain rare.^29^ Several approaches have thus been developed to increase the proportion of patients and cancers that can be treated using ACT.

Tumor-specific T cell clones can be generated from repeated antigen-specific stimulation of patient-derived (autologous) or donor-derived (allogeneic) T cells *in vitro.*^30^ For example, a recent pilot study explored the use of allogeneic CD8+ T cells with activity against the Wilms tumor antigen 1 (WT1) in leukemia patients who relapsed after HSCT.^31^ Clones were generated by leukophoresis of human leukocyte antigen (HLA)-matched donor cells and repeated stimulation with peptide-pulsed, autologous dendritic cells over several months. Adoptively transferred lymphocytes remained detectable in patient blood long-term, and transient responses were observed in 2/11 of these high relapse-risk patients, with stable disease observed in 3 others. Similar approaches have been applied to CTL clones in melanoma^32^,^33^ and ovarian cancer.^34^

A potentially limitless source of T cells with almost any desired specificity can be derived from autologous lymphocytes genetically engineered to express a relevant T-cell receptor (TCR) (reviewed by Kershaw et al.^35^). Tumor-reactive TCR must first be identified in T cells isolated from patients with naturally occurring anti-tumor activity, and can be engineered to increased affinity specificity by changes to complementarity-determining regions.^36^ Antigen-specific TCR may also be derived from mice engineered to express human antigens.^37^ Retroviral or lentiviral transfection is then used to transfer cDNA encoding the desired TCR specificity to T cells isolated from the patient.^38^ A key theoretical concern with this approach is that engineered T cells contain both endogenous and engineered TCR (and thus possess dual specificity), which may lead to cross-reactivity after activation. The TCR chains from native and engineered TCR could also pair to create novel TCR with new specificities. Despite this concern, side effects of genetically engineered T cell therapy have primarily been due to on-target effects of tumor antigens expressed on healthy tissue.

In melanoma, genetically engineered T cells have not been as successful as TIL-derived ACT,^39^ with only 2/18 patients showing partial response and sustained levels of circulating cells at one year. A subsequent study of two additional TCR specificities showed objective response rates of 19%–30%.^37^ However, the use of genetic engineering has vastly expanded the range of cancers potentially amenable to ACT therapy, including neuroblastoma,^40^ synovial cell sarcoma,^41^ and colorectal cancer,^42^ among others. However, the use of TCR derived from responses in other patients both eliminates the contribution of central tolerance and increases the risk of autoimmune toxicity.^41^ Despite the comparatively weak responses and safety concerns, interest in this approach is high due to its ability to treat a variety of cancers and the potential to improve results by additional genetic modifications, as will be discussed later.

While gene-modified T cells can be generated against many tumor antigens, TCR are still HLA-restricted, meaning that new specificities must be described for each tumor antigen and HLA allele. The development of chimeric antigen receptors (CARs) provided a more universal approach to targeting tumor antigens that are expressed on the membrane of cancer cells. Now in their third generation, CARs are hybrid receptors formed by the fusion of an extracellular tumor-specific antibody fragment, a CD3-derived ITAM signaling chain, and a co-stimulatory signaling domain.^43^,^44^ The CARs have been explored with some success against CD19 in B-cell acute lymphoblastic leukemia (B-ALL),^45^ carbonic anhydrase in renal cell carcinoma,^46^ and L1 adhesion molecule (CD171) in neuroblastoma,^47^ among other antigens (reviewed by Kershaw et al.^35^). The most promising results have been achieved in hematologic malignancies,^48^,^49^ where CD19-targeted CARs can recapitulate the mechanism of rituximab therapy without the need for repeated antibody administration.

In summary, there are three possible sources of tumor-specific T cells for adoptive immunotherapy (Figure 2). Tumor-infiltrating lymphocytes, which are naturally enriched for tumor-specific T cells, can be expanded non-specifically *in vitro*, then rein-fused into their original host. For patients in whom TILs cannot be cultured, tumor-specific responses can be derived by antigen-specific expansion or genetic engineering of polyclonal T cell populations. The greatest success has thus far been achieved with TILs, but antigen-specific expansion and genetic engineering are promising approaches to expanding ACT availability.

## COST AND AVAILABILITY

One of the hurdles that must be surpassed for ACT to be generally more relevant to patients with cancer, and not just performed in specialized and subsidized settings, is its economic cost. These massively complex therapies require the development of a “new drug” for each patient, with weeks of cell culture, skilled man-hours, and patient preparation.

Recently, the cancer vaccine sipuleucel-T (Provenge^®^, Dendreon Corporation, Seattle, WA, USA) was approved by the United States Federal Drug Administration for treating prostate cancer.^50^ This approach requires the isolation of immune cells (in this case, primarily dendritic cells) from each individual patient. The cells are then shipped to a central processing lab where they are cultured *in vitro* with tumor antigens and growth factors and are subsequently shipped back to treatment centers of infusion back into the patient, all at a cost of $93,000 per course of treatment.^51^ While sipuleucel-T demonstrated that dendritic cell-based immunotherapy approaches can be effective, the high price and complicated process may limit its long-term benefit to patients with cancer.^52^ Despite the only modest survival benefit conferred by sipuleucel-T, this procedure has been watched closely as proof of concept that cellular therapies can be both clinically effective and economically viable.

The economic model developed for sipuleucel-T relies on a culture process that is patented and centralized by a single private company. In contrast, adoptive T cell therapy for melanoma and other solid tumors has thus far been conducted primarily at academic medical centers and reimbursed via funds set aside for research trials and hospital payments. In this sense, the reimbursement model most closely resembles hematopoietic stem cell transplantation (HSCT), the earliest form of adoptive immunotherapy, which is widely used and reimbursed despite its high cost. In the United States and several other countries, reimbursement is based on a diagnosis-related group (DRG), a bundled payment for the wide range of costs that may be incurred during a single hospitalization for HSCT. The DRG-based reimbursement depends on the development and approval of treatment-specific reimbursement codes, which necessarily lag behind scientific developments and can lead to confusion regarding the ultimate level of reimbursement for a given hospitalization.^53^

As of 2012, the costs of an initial hospitalization for autologous transplantation in the United States ranged from $36,000 to $88,000, while the costs of allogeneic transplantation ranged from $96,000 to $204,000.^54^ These costs varied significantly with patient characteristics, the experience and efficiency of the transplant center, and the protocol used. Furthermore, they were calculated by accounting for products and provider services utilized during hospitalization for transplantation, and thus do not necessarily take into account all ancillary procedures required for HSCT (such as donor matching or long-term follow-up), nor do they represent the charges billed to payers above and beyond the reported costs. Thus, the true costs are difficult to define. Similarly, costs and charges for adoptive T cell therapy are difficult to estimate, as research protocols are constantly changing and, in the context of research trials, are not necessarily publicly reported.

Given these high costs, could adoptive T cell therapy ever be considered cost-effective, particularly in comparison to existing therapies? Unfortunately, it is too early to tell. Cost-effectiveness is dependent not only on the costs associated with ACT, which are currently in flux, but also on the clinical benefit attained by these therapies. In fact, it may be the latter parameter which is most important. While the process of ACT is complex and expensive, it can at least theoretically confer the patient with immune memory that leads to sustained responses for many years. Thus, while any given infusion cycle of a drug will seem comparatively cheap, an expensive ACT procedure that confers long-term benefit could be economically viable.

## THE FUTURE OF ADOPTIVE T CELL IMMUNOTHERAPY

There is no longer any doubt that, under optimal circumstances and for certain cancers, ACT *can* induce regression of established tumors. This has been shown in a number of malignancies ranging from melanoma to certain types of leukemia, as well as prostate cancer. While initial studies and results are very encouraging and provide proof-of-principle evidence, several aspects of ACT need to be optimized. Current efforts focus on expanding affordability and availability. This ultimately depends on a source of tumor-specific T cells that can be generated cheaply, reliably, and quickly.

## Abbreviations:

ACT: adoptive T cell therapy;
B-ALL: B-cell acute lymphoblastic leukemia;
DRG: diagnosis-related group;
HLA: human leukocyte antigen;
HSCT: hematopoietic stem cell transplantation;
MHC: major histocompatibility complex;
NCI: National Cancer Institute;
PBLs: peripheral blood lymphocytes;
TAAs: tumor-associated antigens;
TCR: T-cell receptor;
TILs: tumor-infiltrating lymphocytes.
